# The chaperone HSPB8 reduces the accumulation of truncated TDP-43 species in cells and protects against TDP-43-mediated toxicity

**DOI:** 10.1093/hmg/ddw232

**Published:** 2016-07-27

**Authors:** Valeria Crippa, Maria Elena Cicardi, Nandini Ramesh, Samuel J. Seguin, Massimo Ganassi, Ilaria Bigi, Chiara Diacci, Elena Zelotti, Madina Baratashvili, Jenna M. Gregory, Christopher M. Dobson, Cristina Cereda, Udai Bhan Pandey, Angelo Poletti, Serena Carra

**Affiliations:** 1 Genomic and post-Genomic Center, C. Mondino National Institute of Neurology Foundation, 27100 Pavia, Italy; 2 Dipartimento di Scienze Farmacologiche e Biomolecolari (DiSFeB), Centro di Eccellenza sulle Malattie Neurodegenerative, Università degli Studi di Milano, 20133 Milano, Italy; 3 Department of Pediatrics, Division of Child Neurology, Children’s Hospital of Pittsburgh, University of Pittsburgh Medical Center, Pittsburgh, PA 15224, USA; 4 Department of Human Genetics, University of Pittsburgh Graduate School of Public Health, Pittsburgh, PA 15261, USA; 5 Department of Biomedical, Metabolic and Neuronal Sciences, University of Modena and Reggio Emilia, 41125 Modena, Italy; 6 Department of Cell Biology, University Medical Center Groningen, Groningen, The Netherlands; 7 Department of Chemistry, University of Cambridge, Lensfield Road, Cambridge CB2 1EW, UK

## Abstract

Aggregation of TAR-DNA-binding protein 43 (TDP-43) and of its fragments TDP-25 and TDP-35 occurs in amyotrophic lateral sclerosis (ALS). TDP-25 and TDP-35 act as seeds for TDP-43 aggregation, altering its function and exerting toxicity. Thus, inhibition of TDP-25 and TDP-35 aggregation and promotion of their degradation may protect against cellular damage. Upregulation of HSPB8 is one possible approach for this purpose, since this chaperone promotes the clearance of an ALS associated fragments of TDP-43 and is upregulated in the surviving motor neurones of transgenic ALS mice and human patients. We report that overexpression of HSPB8 in immortalized motor neurones decreased the accumulation of TDP-25 and TDP-35 and that protection against mislocalized/truncated TDP-43 was observed for HSPB8 in *Drosophila melanogaster*. Overexpression of HSP67Bc, the functional ortholog of human HSPB8, suppressed the eye degeneration caused by the cytoplasmic accumulation of a TDP-43 variant with a mutation in the nuclear localization signal (TDP-43-NLS). TDP-43-NLS accumulation in retinal cells was counteracted by HSP67Bc overexpression. According with this finding, downregulation of HSP67Bc increased eye degeneration, an effect that is consistent with the accumulation of high molecular weight TDP-43 species and ubiquitinated proteins. Moreover, we report a novel *Drosophila* model expressing TDP-35, and show that while TDP-43 and TDP-25 expression in the fly eyes causes a mild degeneration, TDP-35 expression leads to severe neurodegeneration as revealed by pupae lethality; the latter effect could be rescued by HSP67Bc overexpression. Collectively, our data demonstrate that HSPB8 upregulation mitigates TDP-43 fragment mediated toxicity, in mammalian neuronal cells and flies.

## Introduction

Amyotrophic lateral sclerosis (ALS) is the most common adult-onset motor neurone disease, which leads to progressive muscle weakness and eventual respiratory failure. ALS is a complex disease that develops in familial (fALS) forms, associated with specific gene mutations in 5–10% of the cases and in sporadic (sALS) forms in the remaining 90% of patients (1). The vast majority of ALS patients show mislocalization and accumulation of the TAR-DNA-binding protein 43 (TDP-43) in affected tissues. Accumulation of TDP-43 is also evident in other neurodegenerative diseases such as frontotemporal lobar degeneration with ubiquitin-positive inclusions (FTLD-U) and Alzheimer's disease (AD) (2–4).

TDP-43 is a predominantly nuclear protein that belongs to the family of heterogeneous nuclear ribonucleoproteins (hnRNPs) and functions in RNA processing (RNA splicing and microRNA biogenesis (5,6)). TDP-43 possesses two RNA recognition motifs (RRMs), a nuclear localization sequence (NLS), a nuclear export signal (NES) and a C-terminal glycine-rich domain. Upon cleavage by caspases at intrinsic caspase cleavage sites, TDP-43 generates two major (but not exclusive (3,7,8)) C-terminal fragments (CTF): the 25 kDa TDP-43 (TDP-25) and the 35 kDa TDP-43 (TDP-35) (7). The two fragments differ in the context that TDP-35 retains both the RRM1 and RRM2 sequences, while only the RRM2 is preserved in TDP-25. It is not clear if this difference can differentially affect TDP-25 and TDP-35 activity, although it has been suggested that only TDP-35 is also able to deregulate pre-RNA splicing (5,9). Both TDP-25 and TDP-35 fragments lack the NLS and retain the NES, thus both mislocalize to the cytoplasm and both are highly aggregation-prone (10).

In addition, experiments in several different cellular models have shown that both TDP-25 and TDP-35 form detergent-insoluble ubiquitin-positive cytoplasmic inclusions. These inclusions sequester endogenous TDP-43 (11) and correlate with reduced TDP-43 levels in the nucleus (12). These TDP-43 aggregates have been documented in almost all cases of sALS and fALS, except for SOD1-linked fALS, and are characterized by the invariable presence of phosphorylated forms of full-length (FL) and fragmented TDP-43; thus TDP-43 aggregation is considered as a pathological signature for ALS (3,7). The seeding by the TDP-25 fragment seems to be required to generate insoluble FL TDP-43-positive aggregates in the cytoplasm (7). TDP-43 and its fragments can be degraded by all intracellular degradative systems (i.e. by the proteasome, macroautophagy, and also by chaperone-mediated autophagy/CMA), but the proteasome-mediated degradation appears to be the preferred route for its disposal (7). In ALS and FTLD-U, when the proteasome is impaired (or overwhelmed) the TDP-35 species accumulate at higher levels than the TDP-25 species, indicating that the two fragments may have different clearance mechanisms, aggregation properties and/or toxicity (13).

Independently of which specific form is more aggregation-prone or toxic, since inclusions formed by truncated TDP-25 and TDP-35 cleavage fragments are associated with enhanced cellular toxicity, all approaches aimed at decreasing their aggregation rate and facilitating their disposal may represent successful strategies to counteract motor neurone degeneration in ALS. One way of achieving this objective could be to boost the protein quality control (PQC) system, which survey protein folding/aggregation and assist protein clearance. Indeed, we have already demonstrated that upregulation of the small heat shock protein HSPB8 (a member of the mammalian sHSP/HSPB family) (14), might represent a possible approach mitigating the accumulation of TDP fragments. In particular, in motor neurone cells HSPB8 decreases the aggregation and enhances the autophagy-mediated degradation of a C-terminal truncated fragment of TDP-43 (ΔC-TDP-43) originating from the frameshift mutation p.Y374X (15,16), while its pharmacological upregulation reduces the accumulation of all species deriving from FL TDP-43 and from the TDP-25 fragment (17). In addition, HSPB8 levels are upregulated in the motor neurones that survive in the spinal cord (15) and affected muscles (18) of transgenic G93A-SOD1 mice, as well as in the spinal cord of ALS patients (19). Finally, HSPB8 mutations (K141E and K141N) have been associated with motor neurone diseases (20–23), further strengthening the hypothesis that alteration of HSPB8 function could have a significant impact on motor neurone viability and that its potentiation/upregulation could help protect against disease.

Based on these findings, we have studied the accumulation and aggregation of TDP-25 and TDP-35 in mammalian motor neurone cells and in the model organism *Drosophila melanogaster* (Dm). We find that overexpression of HSPB8 and its Dm functional ortholog HSP67Bc (24) can significantly decrease truncated and mutant TDP-43 mediated aggregation in cells and eye degeneration in fruitfly, respectively.

## Results

### Evaluation of the biochemical behaviour of GFP-tagged FL TDP-43 and of the two GFP-tagged fragments TDP-35 and TDP-25 in immortalized motor neurone cells

**Figure 1.**
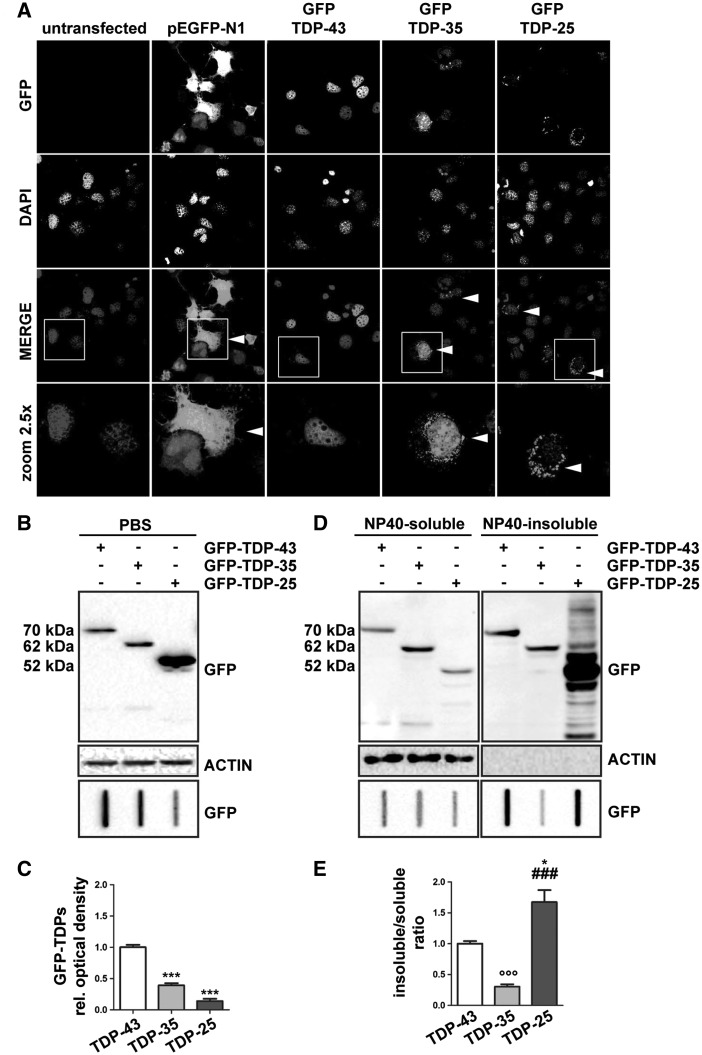
GFP-tagged truncated TDP-35 and TDP-25 show different biochemical behaviour from that of full-length TDP-43. (**A**) Confocal microscopy analysis (63× magnification) of mouse motor neurone NSC34 cells transiently overexpressing GFP-TDP-43, GFP-TDP-35, GFP-TDP-25, or GFP alone (pEGFP-N1, used as a control). DAPI: nuclei staining. A 2.5x magnification of selected areas is shown. (**B**): Western Blot (WB; upper insets) and filter retardation assay (FRA, lower inset) of the PBS extracts of mouse motor neurone NSC34 cells transiently overexpressing the GFP-TDP-43, GFP-TDP-35 or GFP-TDP-25. (**C**) Quantification of the PBS extract by FRA (***P < 0.001 vs GFP-TDP-43). The data represent in each case the mean ± sem of *n = *3 independent samples. (**D**): WB (upper insets) and FRA (lower insets) of the NP40 extracts of cells transiently overexpressing GFP-TDP-43, GFP-TDP-35 or GFP-TDP-25 (left insets, NP40-soluble fraction; right insets, NP40-insoluble fraction). (**E**): Quantification of FRA NP40 insoluble/soluble ratio (°°°*P < *0.001 vs GFP-TDP-43; * *P < *0.05 vs GFP-TDP-43; ### *P < *0.001 vs GFP-TDP-35). The data again represent the mean ± sem of *n*=3 independent samples.

We characterized the biochemical behaviour of the GFP-tagged FL TDP-43 and the two major fragments derived from its caspase cleavage TDP-35 and TDP-25 in immortalized motor neurone NSC34 cells. By transiently overexpressing the different N-terminal GFP-tagged TDP variants, or GFP itself (using pEGFP-N1 plasmid), we found that, while GFP was distributed in a diffuse manner, GFP-TDP-43 was confined to the nucleus in the vast majority of motor neurones, while both GFP-TDP-35 and GFP-TDP-25 were observed to be mislocalized to the cytoplasm (Figure 1A). Cells transfected with GFP-TDP-25 formed aggregates in a large fraction of cases (62.7 ± 13.7% of cells) compared with those transfected with GFP-TDP-43 (8.0 ± 4.9% of cells) and GFP-TDP-35 (30.0 ± 14.1% of cells) (Figure 1A). The GFP-TDP-25 aggregates appeared as large rounded inclusions localized and dispersed in the cell cytoplasm, while GFP-TDP-35 had accumulated both in the nucleus, where it was characterized by a diffuse pattern and in the cytoplasm, where it formed aggregates (Figure 1A). We next analysed the solubility properties of the different TDP variants in immortalized motor neurones using two techniques: western blot (WB) and filter retardation assay (FRA). While the WB assay is designed for detection of the protein in its monomeric form solubilized by SDS, as well as SDS-resistant oligo-heteromeric species, FRA allows the measurement of the PBS-resistant TDP aggregates accumulating in the same samples. The WB data showed that the levels of monomeric SDS-soluble GFP-TDP-25 were much higher than those of GFP-TDP-43 and GFP-TDP-35, whose levels were comparable to each other (Figure 1B, upper panel). In contrast, by FRA we observed that the levels of PBS-resistant aggregated GFP-TDP-43 were much higher than those of GFP-TDP-35 and GFP-TDP-25 (Figure 1B, lower inset and 1C, quantification bar graph). This finding is in contrast to our immunofluorescence data demonstrating a high tendency of GFP-TDP-25 to form cytoplasmic aggregates and suggests that detergents are required to extract and solubilize at least some fractions of aggregated GFP-TDP-25 and GFP-TDP-35. Indeed, when using a lysis buffer containing the detergent NP40, we could detect a significant accumulation of GFP-TDP-25 in the NP40-insoluble fraction, while the levels of NP40-soluble GFP-TDP-43, GFP-TDP-35 and GFP-TDP-25 were all similar (Figure 1D and E). Thus, the aggregates visible by immunofluorescence are most likely to correspond to NP40-insoluble GFP-TDP-25 species. The data obtained using NP40 lysis buffer and immunofluorescence, however, reveal that TDP-25 and TDP-35 fragments have different aggregation properties, with the TDP-35 fragment being much more soluble than the TDP-25 fragment.

### The role of the degradative systems on the solubility of GFP-tagged FL TDP-43 and of the two GFP-tagged fragments TDP-35 and TDP-25 in mammalian motor neurone cells

**Figure 2.**
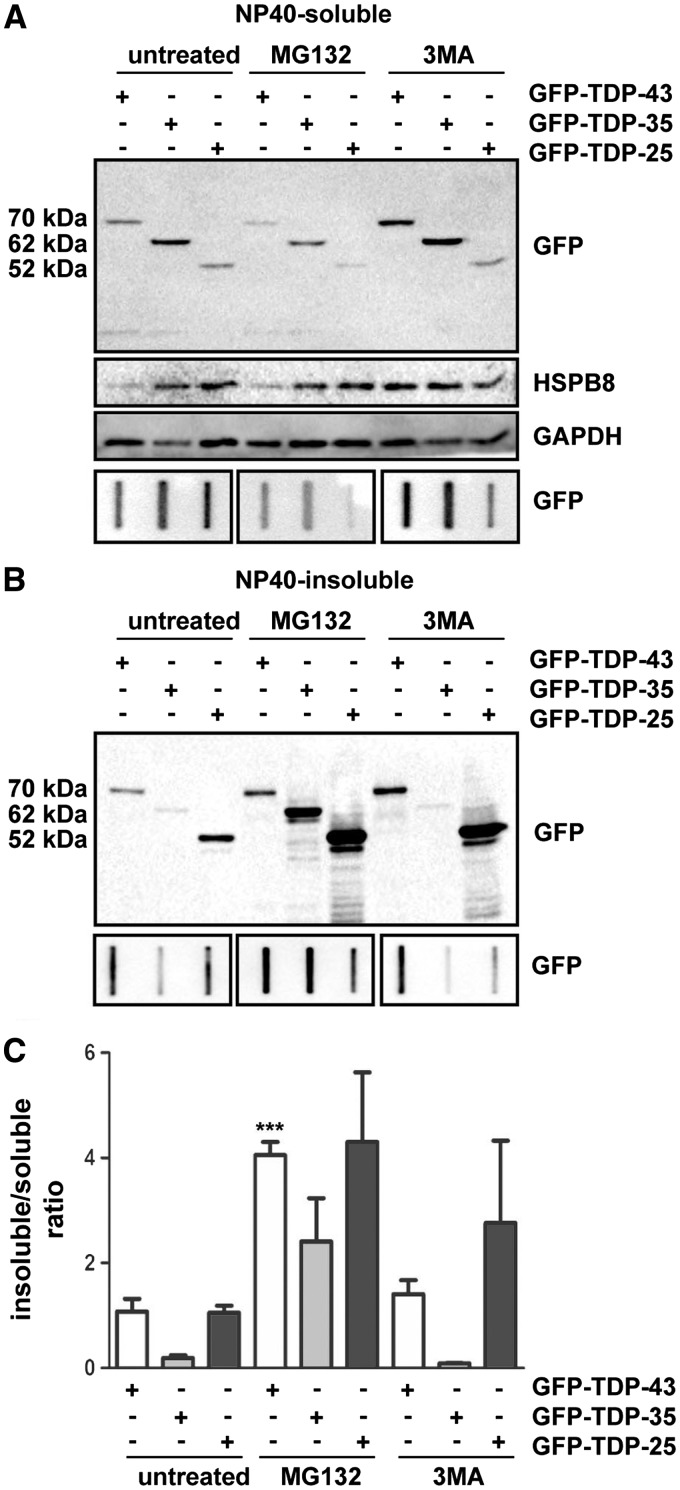
GFP-TDP-35 and GFP-TDP-25 show different clearance from that of full-length GFP-TDP-43. (**A**) WB (upper insets) and FRA (lower insets) of the NP40-soluble extracts of mouse motor neurone NSC34 cells transiently overexpressing GFP-TDP-43, GFP-TDP-35 or GFP-TDP-25 and treated with 10 µM MG132 for 16 h or 10 mM 3-methyladenine (3-MA) for 32 h. (**B**) WB (upper insets) and FRA (lower insets) of NP40-insoluble extracts. (**C**) Quantification of FRA NP40 insoluble/soluble ratios (***P < 0.001 vs GFP-TDP-43). The data represent the mean ± sem of *n*=3 independent samples.

The aggregation propensity of TDP-25 versus TDP-43 and TDP-35 might depend not only on the intrinsic properties of these different species but also on the ability of the cell to clear them. If differences in the turnover of the cleaved TDP-43 fragments exist, their clearance should be differentially affected when the degradative systems are pharmacologically impaired. Therefore, we analysed how inhibition of the proteasome and of autophagy impacts on the intracellular levels of the different GFP-TDP variants. In this assay, we use the NP40 fractionation method, which, according to our results, is the most suitable method to analyse the accumulation of GFP-TDP aggregates. Inhibition of autophagy or of the proteasome system mildly increased the levels of the NP40-soluble monomeric forms of GFP-TDP-43 and GFP-TDP-35, while leaving monomeric NP40-soluble GFP-TDP-25 unaffected (Figure 2A). In contrast, monomeric NP40-insoluble (SDS-soluble) GFP-TDP-25 accumulated significantly in immortalized motor neurones after inhibition of both the proteasome and autophagy (Figure 2B). A small increase in the levels of monomeric NP40-insoluble (SDS-soluble) GFP-TDP-35 was detected exclusively after proteasome inhibition, while no changes were observed in the case of GFP-TDP-43 (Figure 2B and C). These data suggest that all the GFP-TDP variants can be processed by the proteasome and tend to accumulate when this degradative pathway is overwhelmed and/or impaired, while the levels of the TDP-25 fragment, which has a higher aggregation propensity also depends on a functional autophagic flux for proper clearance. This finding is in line with the role of autophagic vacuoles in the engulfment and degradation of proteinaceous aggregates and with the experimental evidence that autophagy efficiently clears aggregates, protecting against misfolded protein mediated toxicity (25,26).

### The effects of HSPB8 on the solubility and clearance of GFP-tagged FL TDP-43 and of the two GFP-tagged fragments TDP-35 and TDP-25 in immortalized motor neurone cells

**Figure 3.**
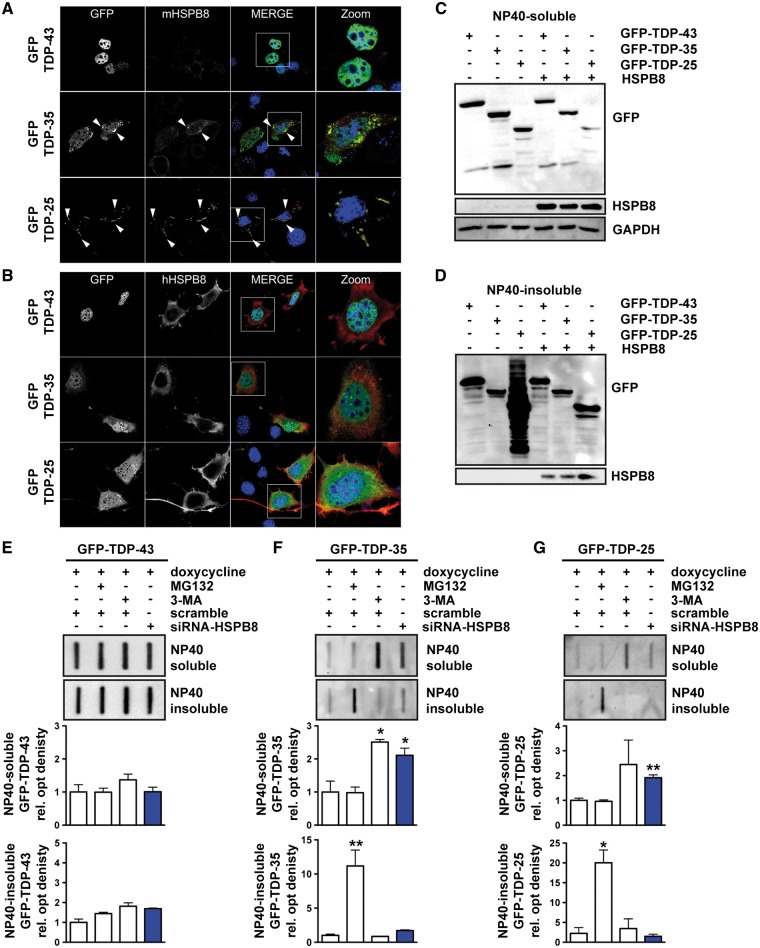
HSPB8 affects the accumulation of GFP-TDP-35 and GFP-TDP-25 fragments. (**A,B**) Confocal microscopy analysis (63× magnification) of NSC34 cells transiently co-transfected with plasmids coding for GFP-TDP-43, GFP-TDP-35 or GFP-TDP-25 and pCDNA3 (A) or hHSPB8 plasmid (B). DAPI: nuclei staining. RED: murine endogenous HSPB8 (mHSPB8) or human transfected HSPB8 (hHSPB8). A 2.5× magnification of selected areas is shown. (**C**) WB of the NP40-soluble extracts of mouse motor neurone NSC34 cells transiently overexpressing GFP-TDP-43, GFP-TDP-35 or GFP-TDP-25 and human HSPB8. (**D**): WB of the NP40-insoluble extracts. E-G: Representative FRA (upper panels) and FRA quantification (lower panels) of the NP40-soluble and NP40-insoluble extracts of mouse motor neurone NSC34 cells stably overexpressing GFP-TDP-43 (**E**), GFP-TDP-35 (**F**) or GFP-TDP-25 (**G**), induced for 72 h with 1 μg/ml doxycycline and transfected with scramble or siRNA-HSPB8; the cells were treated with 10 μM MG132 and 10 mM 3-MA for the last 16 h prior to extraction. The optical densities of GFP-TDPs were expressed relative to the mean optical densities of the corresponding samples treated with doxycycline and a scramble RNAi sequence, taken as internal references. The data represent the mean ± sem of *n = *3 independent samples. (*P < 0.05 vs scramble; ** *P < *0.01 vs scramble).

The observation that HSPB8 expression is enhanced by the expression of both GFP-TDP-35 and GFP-TDP-25 fragments (Figure 2A, WB lanes 2 and 3), prompted us to evaluate the effect of this chaperone on the three TDP variants. We previously reported that HSPB8 enhances the autophagy-mediated clearance of ΔC-TDP-43, the N-terminal fragment of TDP-43 (15,16), found in some patients affected by fALS (frameshift mutation p.Y374X) (27). In addition, HSPB8 has been found to be upregulated in the surviving motor neurones in ALS mice and in ALS patients, supporting the view that this chaperone may exert a beneficial effect on motor neurone survival (15). Intriguingly, as stated above, HSPB8 was significantly upregulated in NSC34 cells transfected with GFP-TDP-35 and GFP-TDP-25 as compared to those transfected with GFP-TDP-43 (Figure 2A, untreated cells), which may represent a response within the cells to facilitate the clearance of these aberrant TDP species. Interestingly, we found that endogenous HSPB8 (mHSPB8) co-localized with the large GFP-TDP-35 inclusions (Figure 3A, arrows) and with all the GFP-TDP-25 aggregates (Figure 3A, arrows). Overexpression of human HSPB8 (hHSPB8) in NSC34 cells transiently transfected with the three different TDP variants almost completely abrogated GFP-TDP-35 and GFP-TDP-25 aggregation, with a parallel increase of the soluble TDP forms (Figure 3B). The anti-aggregation function of HSPB8 on different TDP species was further investigated by WB. In line with our immunofluorescence results, HSPB8 overexpression slightly decreased the monomeric NP40-soluble levels of all TDP species (Figure 3C), while it significantly inhibited the accumulation of NP40-insoluble GFP-TDP-25 (Figure 3D).

To probe further, the importance of HSPB8 for the degradation of GFP-TDP fragments we performed silencing experiments in inducible stably-transfected GFP-TDPs-NSC34 cell lines that expressed comparable levels of GFP-TDPs mRNA (data not shown). In these cells, HSPB8 silencing resulted in a significant increased accumulation of both GFP-TDP-35 and GFP-TDP-25 NP40-soluble species retained by cellulose acetate membranes (Figure 3 E–G). Consistent with our previous results, inhibition of the proteasome and of autophagy leads to accumulation of GFP-TDP-35 or GFP-TDP-25, but leaves GFP-TDP-43 unaffected (Figure 3 E–G). Taken together, these results support the conclusion that the protein quality control system participates in the clearance of truncated TDP-35 and TDP-25 and highlight HSPB8 as one of the critical players required for this action.

### In immortalized motor neurone cells, FLAG-tagged TDP-35 and TDP-25 mislocalize to the cytosol and are cleared in a proteasome and autophagy-dependent manner

**Figure 4.**
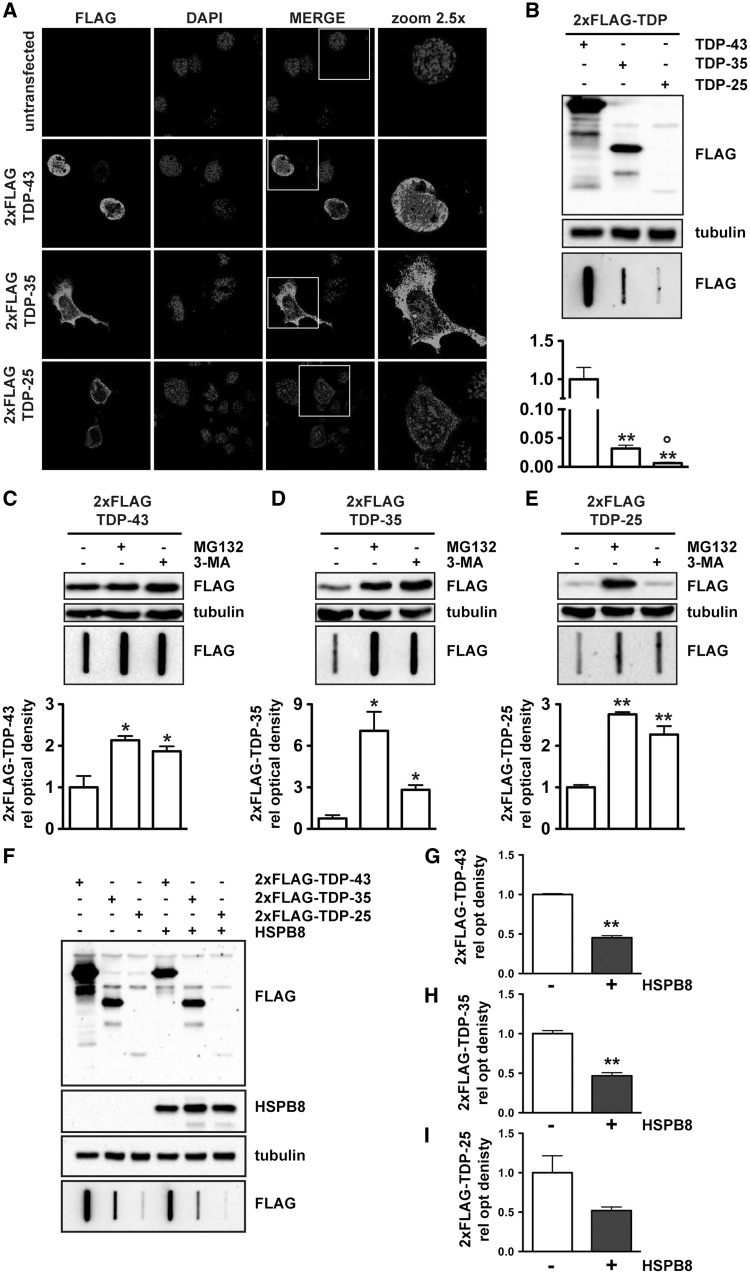
Accumulation and clearance of FLAG-tagged TDP-43, TDP-35 and TDP-25. (**A**) Confocal microscopy analysis (63× magnification) of mouse motor neurone NSC34 control cells or cells transiently overexpressing 2xFLAG-TDP-43, 2xFLAG-TDP-35, 2xFLAG-TDP-25. DAPI: nucleic acid staining. A 2.5x magnification image of selected areas is shown. (**B**) WB (upper insets) and FRA (lower insets and FRA quantification, bar graph) of the PBS extracts of mouse motor neurone NSC34 cells transiently overexpressing the 2xFLAG-TDP-43, 2xFLAG-35 or 2xFLAG-TDP-25. The data represent the mean ± sem of *n = *3 independent samples. ** *P < *0.001 vs 2xFLAG-TDP-43; ° *P < *0.05 vs 2xFLAG-TDP-35. (**C-E**): WB (upper insets) and FRA (lower insets and FRA quantification, bar graph) of the PBS extracts of mouse motor neurone NSC34 cells transiently overexpressing 2xFLAG-TDP-43 (C), 2xFLAG-35 (D) or 2xFLAG-TDP-25 (E) and treated with 10 µM MG132 for 16 h or 10 mM 3-methyladenine (3-MA) for 32 h (**P < 0.001 or * *P < *0.01 vs untreated controls). The data represent the mean ± sem of *n = *3 independent samples. (**F-I**): NSC34 cells transiently co-transfected with plasmids coding for 2xFLAG-TDP-43, 2xFLAG-TDP-35 or 2xFLAG-TDP-25 and pCDNA3 or hHSPB8. WB and representative FRA (F). FRA quantification (G, TDP-43; H, TDP-35; I, TDP-25) (**P < 0.001 vs pCDNA3 transfected cells). The data represent the mean ± sem of *n*=3 independent samples.

As the GFP tag has the potential to influence the stability, distribution and/or aggregation properties of truncated TDP-43, we repeated our experiments using a FLAG tag instead of GFP; the smaller size of the former is likely to reduce any perturbation effects. We therefore produced N-terminal FLAG-tagged TDP variants (2xFLAG-TDP-43, 2xFLAG-TDP-35 and 2xFLAG-TDP-25), and characterized their biochemical behaviour after transient overexpression in immortalized motor neurone NSC34 cells. Similarly to the GFP-tagged TDPs described above, 2xFLAG-TDP-43 was found to be confined to the nucleus with very few cells forming aggregates (7.14 ± 1.36% of cells), while both 2xFLAG-TDP-35 and 2xFLAG-TDP-25 were mislocalized into the cytoplasm (Figure 4A). However, while GFP-TDP-35 and GFP-TDP-25 formed cytosolic insoluble aggregates, FLAG-TDP-35 and FLAG-TDP-25 accumulated at much lower levels and were mainly diffuse in appearance, with only small aggregates visible in ca 20% of transfected cells (20.0 ± 2.53 and 19.80 ± 2.80% of cells, respectively), suggesting that FLAG-TDP-35 and FLAG-TDP-25 could be rapidly degraded by the cellular machineries. In line with this interpretation, we found that in resting cells the soluble and insoluble levels of FLAG-TDP-35 and, particularly of FLAG-TDP-25, were much lower than those of FLAG-TDP-43. However, both soluble and insoluble FLAG-TDP-35 and FLAG-TDP-25 accumulated to very significantly greater levels upon inhibition of the proteasome and of autophagy, supporting the conclusion that they are targeted for degradation by both systems (Figure 4C–E). Taken together, these results highlight the fact that FLAG-TDP-35 and FLAG-TDP-25 have different aggregation propensities from those of the GFP-tagged forms (compare Figure 4C–E with Figure 3E–G); thus caution must be taken when using large tags on such small proteins. However, the results also demonstrate that TDP-43 and its truncated forms are processed preferentially both by the proteasome and by autophagy, regardless of the type of tag that was employed.

### HSPB8 decreases the accumulation of FLAG-tagged TDP-35 and TDP-25 in immortalized motor neurone cells

We next evaluated the effects of overexpression of HSPB8 on the intracellular distribution and accumulation of the 2xFLAG-tagged TDP-43, TDP-35 and TDP-25. Similarly to the observation made using GFP-tagged TDP-43, TDP-35 and TDP-25, co-expression of HSPB8 decreased the accumulation of both soluble and insoluble species of all 2xFLAG-tagged TDP variants as measured by WB (Figure 4F) and FRA (Figure 4F, lower inset and Figure 4G–I). A decrease in the accumulation of soluble and aggregated TDP-43 species by co-expression with HSPB8 was further confirmed by confocal microscopy (Supplementary Material, Fig. S1). In combination, these results further suggest that HSPB8 participates in the disposal of accumulating and/or mislocalized TDP43 variants independently on the type of tag used.

### Overexpression of HSP67Bc in flies reduces the accumulation and toxicity of TDP-43-NLS

The *Drosophila melanogsater* (Dm) family of sHSPs comprises 11 members that have specific functions in the control of protein homeostasis (28). We have previously identified HSP67Bc as the Dm functional ortholog of human HSPB8 (24) and shown that it promotes the autophagic flux in *Drosophila* S2 cells, facilitating the degradation of a variety of misfolded proteins (24). This pro-degradative effect of HSP67Bc has been shown, for example, to result in a decrease of the total levels of mutated Ataxin 3, both in cells and in *Drosophila* where it protects against eye degeneration (24,28). Based on these findings, we asked whether or not overexpression of HSP67Bc in fruit flies could protect against the toxicity mediated by mutated TDP-43.

**Figure 5.**
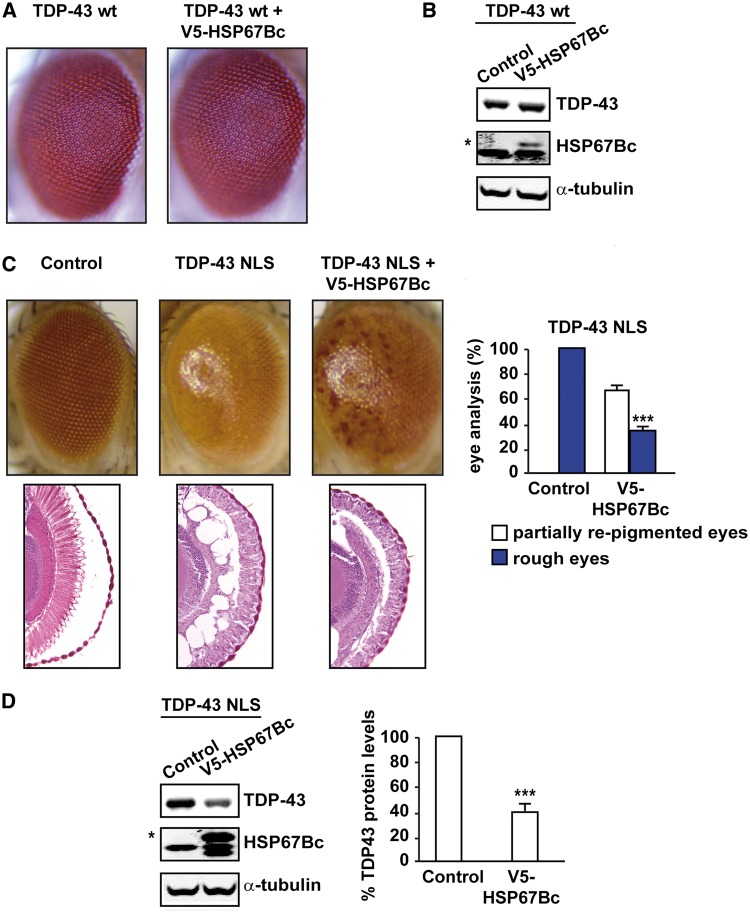
HSP67Bc rescues mutant eye degeneration mediated by TDP-43-NLS. (**A**) Representative pictures showing eyes from 1-day-old flies expressing human TDP-43 under the control of the gmr-GAL4 driver alone (TDP-43 wt: GMR-TDP-43 wt/w1118) or with V5-HSP67Bc. (**B**) WB showing expression levels of TDP-43 and V5-HSP67Bc (*corresponds to V5-tagged HSP67Bc). α-tubulin was used as the loading control. (**C**) Top row, Representative pictures showing eyes from control flies (Control: GMR-GAL4/w1118) or flies expressing human NLS-mutant TDP-43 under the control of the gmr-GAL4 driver alone (TDP-43 NLS: GMR-TDP-43 NLS/w1118) or with V5-HSP67Bc. The eyes of flies expressing mutant TDP-43 NLS show partial loss of ommatidia, a rough phenotype and depigmentation, while co-expression with HSP67Bc rescues the eye pigmentation and ommatidia organization. Bottom row, corresponding Richardson's stained frontal sections showing mutation-dependent internal degeneration that is rescued by overexpression of HSP67Bc. The quantification of the extent of eye degeneration (partially re-pigmented eyes and rough eyes) is reported (***P<0.001; V5-HSP67Bc expressing flies versus control flies). Between 91 and 126 eyes/fly genotype were scored (*n = *3 ± sem). (**D**) WB showing expression levels of mutant TDP-43 NLS and HS67Bc in the eyes of 1-day-old flies. α-tubulin served as the loading control. The quantification of TDP-43 NLS protein levels is shown (***P<0.001; V5-HSP67Bc expressing flies versus control flies). For the analysis of TDP-43-NLS protein, *n = *3–5 independent samples ± sem; each sample was obtained from 10 to 12 fly heads.

Ubiquitous overexpression of both WT TDP-43 and a mutant lacking the nuclear localization signal (NLS) have been shown to result in premature lethality in flies (29). This finding is consistent with the observation that the accumulation of TDP-43 in the cytoplasm of cultured cells, generated by mutating key residues in TDP-43 NLS (30) or by deleting the entire NLS (31), leads to aggregation and toxicity. We first confirmed the finding that the ubiquitous overexpression of TDP-43 in flies leads to premature lethality, and thus we restricted the expression of TDP-43 in the fly eyes. In this study, we used transgenic fruit flies (here referred to as TDP-43-NLS) that express a mutation variant of TDP-43 in which Lys95, Lys97 and Arg98 are all substituted by alanine residues; this variant has previously been characterized for its toxicity in cells (30) and fly eyes (32). As control, we also used the well-characterized fly line expressing WT TDP-43 in the eyes. In agreement with published data (28), overexpression of WT TDP-43 in the fly eyes caused a modest degree of degeneration, referred to as a rough eye phenotype (Figure 5A). Overexpression of HSP67Bc in fly eyes did not by itself affect eye morphology ((24) and Supplementary Material, S2A) and co-expression of HSP67Bc with WT TDP-43 did not significantly affect the eye phenotype caused by WT TDP-43, nor its expression levels (Figure 5A,B). By contrast, expression in fly eyes of NLS-mutant TDP-43 led to a strong degenerative phenotype, characterized by rough and depigmented eyes, accompanied by strong ommatidia disorganization (Figure 5C). Both the eye degeneration and ommatida disorganization were significantly rescued by co-expression of HSP67Bc with NLS-mutant TDP-43 (Figure 5C), finding that correlates with a significant decrease in the total NLS-mutant TDP-43 protein levels as a result of the presence of HSP67Bc (Figure 5D).

The decrease in the extent of eye degeneration and in the total protein levels of mutant TDP-43 NLS observed in flies that co-express HSP67Bc is likely to be due to its pro-degradative function. To explore this issue further, we first used the UAS-eGFP or the UAS-LacZ transgenes to control for the possibility that the differences observed in the absence and presence of HSP67Bc would be due to dilution effects of the GAL4 protein with an increased number of UAS transgenes. We observed no difference in the eye phenotype of flies expressing WT TDP-43 and the TDP-43-NLS mutant alone or with GFP or LacZ (Supplementary Material, S2B and S2C). Second, we co-expressed another member of the *Drosophila* sHSP family, CG14207, which promotes HSP70-dependent protein refolding, with WT and mutant TDP-43-NLS. However, in contrast to HSP67Bc, CG14207 was not able to induce the autophagy-mediated degradation of misfolded proteins, for example of expanded polyglutamine sequences (24,28). Moreover, a previous report has shown that co-expression of CG14207 with WT TDP-43, despite decreasing the extent of its aggregation, did not decrease its total accumulation, further supporting the conclusion that CG14207 does not promote TDP-43 clearance (28). In agreement with these findings, the eye phenotype and the protein levels of TDP-43 were similar in flies co-expressing the WT and NLS-mutant TDP-43 alone or with CG14207 (Supplementary Material, Fig. S3A–F). Taken together, our results suggest that the protective effects on eye degeneration and the reduction of TDP-43-NLS levels in the fly eyes that co-express HSP67Bc are due to its pro-degradative function.

### Downregulation of HSP67Bc in flies increases the accumulation and toxicity of TDP-43-NLS

**Figure 6.**
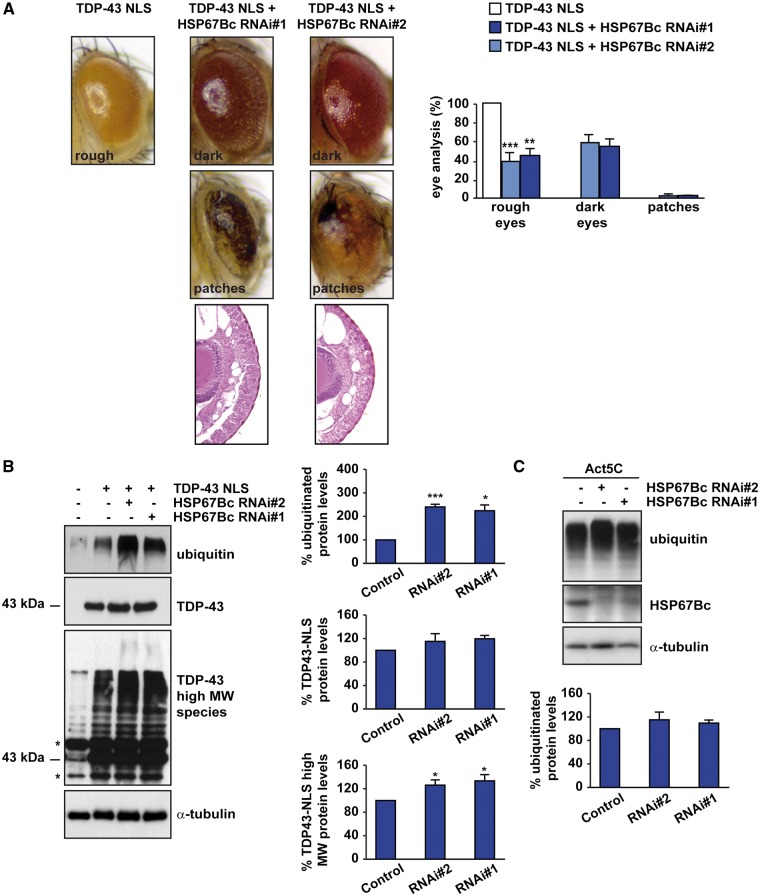
Downregulation of HSP67Bc increases TDP-43-mediated toxicity and impairs protein homeostasis. (**A**) Top and middle row, representative pictures showing eyes from flies expressing human NLS-mutant TDP-43 under the control of the gmr-GAL4 driver alone (TDP-43 NLS: GMR-TDP-43 NLS/w1118) or with two independent sequences silencing endogenous HSP67Bc (RNAi#1 or RNAi#2). Bottom row, corresponding Richardson's stained frontal sections showing internal degeneration in flies with HSP67Bc depletion. The quantification of the extent of eye degeneration (rough eye, dark brown eye or eye with patches) is shown (***P < 0.001; ** *P* < 0.01; HSP67Bc-depleted flies versus control flies). 50–100 eyes/fly genotypes were scored (*n = *3 ± sem). (**B**): WB and data quantitation showing expression levels of mutant NLS TDP-43, high molecular weight (MW) species and endogenous ubiquitin in the eyes of 1-day-old flies. α-tubulin was used as the loading control (***P < 0.001; ** *P* < 0.01; * *P* < 0.05; HSP67Bc-depleted flies versus control). For the analysis of monomeric TDP-43, high molecular weight (MW) TDP-43 and ubiquitinated protein, *n*=3–6 independent samples ± sem; each sample was obtained from 10 to 12 fly heads. (**C**) WB and data quantitation showing expression levels of endogenous ubiquitin and HSP67Bc in the eyes of 1-day-old control flies or flies with ubiquitous expression (Act5C driver) of two independent sequences silencing endogenous HSP67Bc (RNAi#1 or RNAi#2). α-tubulin served as the loading control. *n*=3–5 independent samples ± sem; each sample was obtained from 5 whole flies.

In line with this interpretation of the overexpression data, downregulation of endogenous HSP67Bc by two independent RNAi lines that significantly lower HSP67Bc expression (Figure 6C, Supplementary Material, Fig. S4A and (24)), significantly increased the extent of TDP-43-NLS mediated eye degeneration, with darkening and the appearance of dark patches on the eyes (Figure 6A). As with overexpression of HSP67Bc, we observed that knockdown of endogenous HSP67Bc in the fly eyes did not, by itself, affect eye morphology (Supplementary Material, Fig. S4B; compare with Supplementary Material, Fig. S2A, control flies, and 24). Increased eye degeneration due to the downregulation HSP67Bc was found to correlate with a small but significant increase in the high molecular weight (MW) species typically formed on expression of mutant TDP-43-NLS (as previously described (32)) and also with a highly significant accumulation of endogenous (poly)ubiquitinated proteins (Figure 6B). Interestingly, overexpression in mammalian cells of mutant TDP-43-NLS, but not WT TDP-43, results in the accumulation of high MW species that resemble the ubiquitinated TDP-43 high MW smear observed in ALS and FTLD-U cases (30). Similarly, we find here that TDP-43-NLS also has a higher propensity than WT TDP-43 to form high MW species *in vivo* (Supplementary Material, Fig. S4C); these TDP-43-NLS high MW species accumulate further following depletion of HSP67Bc (Figure 6). Finally, the accumulation of (poly)ubiquitinated proteins was not observed in flies where HSP67Bc expression was ubiquitously downregulated (Figure 6C). Together, these results support the conclusion that HSP67Bc plays an important role in the maintenance of protein homeostasis under proteotoxic stress conditions caused by the presence of TDP-43-NLS.

### Overexpression of TDP-35 in flies leads to pupae lethality that can be rescued by co-expression of HSP67Bc

**Figure 7.**
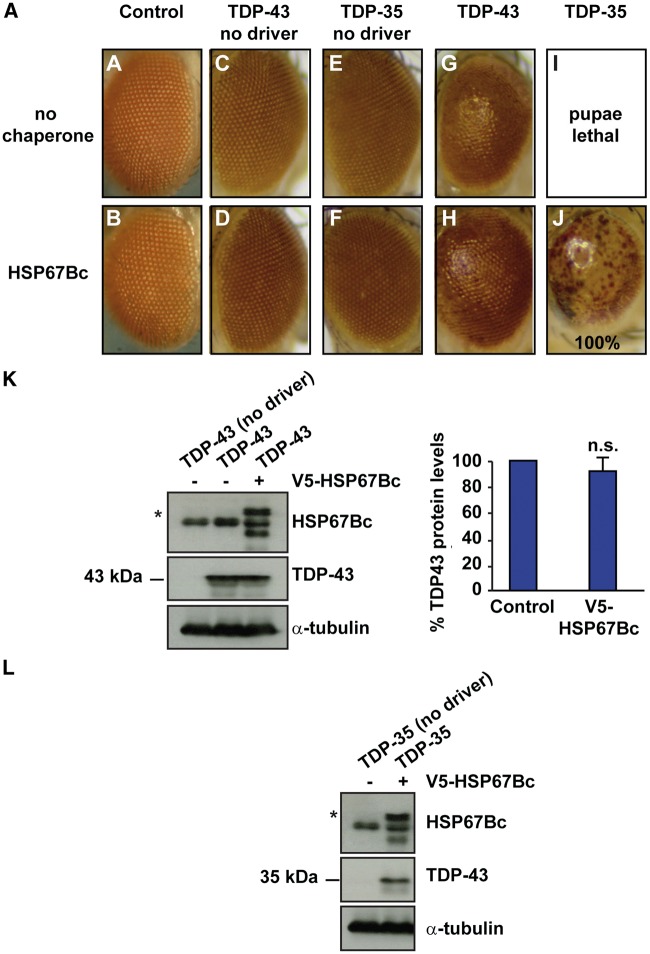
HSP67Bc rescues pupae lethality caused by expression of TDP-35. (**A–J**) Top and bottom row, representative pictures showing eyes from 1-day-old flies expressing human TDP-43 or TDP-35 under the control of the gmr-GAL4 driver alone (no chaperone) or with V5-tagged HSP67Bc (HSP67Bc). Control: gmr/51D (no chaperone) and gmr/51D;HSP67Bc (HSP67Bc alone). For gmr/TDP-35;HSP67Bc (TDP-35/HSP67Bc) flies, ca. 40 eyes were scored from 3 independent experiments. (**K, L**) Western blot showing expression levels of TDP-43 wt (K) or TDP-35 (L) and V5-HSP67Bc in the eyes of 1-day-old flies (* corresponds to V5-tagged HSP67Bc). UAS-TDP-43 fly extracts were used as control. α-tubulin served as the loading control. 10 fly heads/sample were used for the analysis of TDP-43 and TDP-35 protein levels.

TDP-43-NLS is an artificially generated mutant that mimics the toxicity due to cytoplasmic accumulation of TDP-43. To study further the protective role of HSP67Bc against TDP-43 mediated toxicity, we generated another fly model which expresses the truncated form TDP-35 observed in ALS patients. Flies expressing WT TDP-43 were used as a control, and to avoid any artefacts due to differences in transgene expression between TDP-43 or TDP-35, both constructs encoding for TDP-43 or TDP-35 were incorporated at the same genomic locus (33). Furthermore, control flies that do not express TDP-43 or TDP-35 (Figure 7A,C and E) or only express HSP67Bc (Figure 7B,D and F) were observed to have normal eyes. In agreement with our previously published data (33), overexpression of TDP-43 in the fly eyes, alone or with HSP67Bc, caused a mild rough eye phenotype (Figure 7, compare G, expression of TDP-43 alone with H, co-expression of TDP-43 and HSP67Bc), resembling the phenotype obtained using another independent fly model (Figure 5A) (32). Similarly to the finding illustrated in Figure 4B, co-expression of HSP67Bc did not affect the expression levels of TDP-43 (Figure 7K). In addition, expression of TDP-25 in the fly eyes caused a very mild degenerative phenotype, indistinguishable from that caused by WT TDP-43 (33). In contrast, expression of TDP-35 in the fly eyes led to pupae lethality (Figure 7, compare E, negative control, with I, expression of TDP-35), which was rescued by co-expression of HSP67Bc (Figure 7J); the observed eclosion rate for flies co-expressing TDP-35 and HSP67Bc was of ca. 21.4% and the expected eclosion rate is of 25%. We verified by WB that these flies indeed express both TDP-35 and HSP67Bc (Figure 7L). However, 100% of the flies co-expressing TDP-35 and HSP67Bc showed partial eye degeneration with depigmentation as shown in Figure 7J. This result supports the conclusion that the toxicity of the TDP-35 fragment is largely, but not fully, suppressed by the presence of the chaperone HSP67Bc in fruit flies.

### Overexpression of HSP67Bc also protects against the eye degeneration induced by the ALS-causing mutant TDP-43 M337V

**Figure 8.**
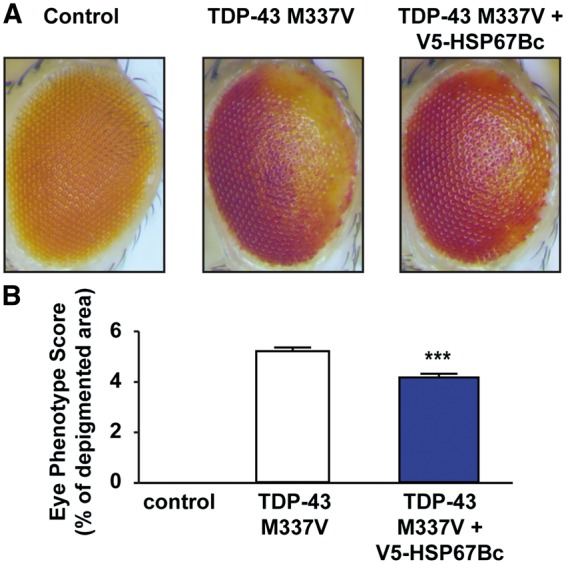
HSP67Bc significantly rescues the eye degeneration induced by the ALS-causing mutant M337V TDP-43. (**A**) Representative pictures showing eyes from 1-day-old control flies (Control: GMR/w1118) or flies expressing human M337V TDP-43 under the control of the gmr-GAL4 driver alone (TDP-43 M337V: GMR-TDP-43 M337V/w1118) or with V5-HSP67Bc. (**B**) Quantitation of the eye phenotype shown in (A). For this experiment the following eye phenotypic score was assessed: eye degeneration score of 4 for area less than 10%; eye degeneration score of 5 for area between 30 and 50%; eye degeneration score of 6 for area between 50 and 70%. In each case 20–39 eyes/fly genotype were scored. ***P < 0.0001.

A number of ALS-linked missense mutations have been identified in the gene encoding for TDP-43, including the M337V mutation (34). We therefore confirmed in the present study that overexpression in the fly eyes of ALS-causing mutant M337V TDP-43 caused neurodegeneration (32), as shown by the partial depigmentation with the loss of ommatidia (Figure 8A and B). To evaluate the extent of eye degeneration, we quantified the area of depigmentation with complete loss of ommatidia. Figure 8 shows that co-expression of HSP67Bc with M337V TDP-43 significantly decreased the area of the eyes characterized by degeneration. These results further support our conclusion that upregulation of HSP67Bc protects against TDP-43 mediated toxicity and show that the protective effects of HSP67Bc extend to various mutant forms of TDP-43, all characterized by mislocalization.

## Discussions

Protein misfolding and aggregation is a common feature of many neurodegenerative disorders, including ALS, FTLD-U, polyglutamine diseases such as Huntington Disease (HD), Parkinson disease (PD) and Alzheimer disease (AD) and decreasing protein aggregation has been shown to be protective in several cellular and animal models of such conditions (18,35–40). Protection from neurodegeneration in such disease models can be achieved by enhancing the degradation of misfolded aggregation-prone species that is mediated by the proteasome or autophagy, or by boosting the expression of molecular chaperones that participate both in protein refolding and protein degradation (15,41–44). These key chaperones include HSPB8, a small heat shock protein that interacts with BAG3, a co-chaperone of HSP70, and that has been shown to facilitate the autophagic removal of a number of aggregation-prone substrates, such as mutated polyglutamine proteins (41,45–47), a mutant SOD1 and a C-terminal truncated fragment of TDP-43 (ΔC-TDP-43) associated with ALS (15,45–47), the β-amyloid peptide associated with AD (48), mutant α-synuclein associated with PD (49), and of other proteins that readily misfold (42,43,50) .

The multi-functional RNA binding protein TDP-43 is emerging as a key pathogenic protein in several neurodegenerative diseases. Indeed, in almost all cases of sporadic and inherited forms of ALS and FTLD-U, protein aggregates have been shown to contain not only ubiquitin, but also TDP-43 (11,51). In ALS and FTLD-U, several post-translational modifications of TDP-43 have been identified, which include hyperphosphorylation and ubiquitination, as well as caspase-mediated proteolysis. TDP-43 cleavage leads to the production of 25 kDa and 35 kDa N-terminally truncated fragments of TDP-43 (referred to as TDP-25 and TDP-35). Virtually all these modifications can contribute to the mislocalization of TDP-43 from the nucleus (where this protein is mainly localized in healthy and resting cells) to the cytosol (where it aggregates), thereby causing the loss of normal TDP-43 function for the modulation of splicing and transcription (11). Moreover, TDP-43 cleavage to produce the C-terminal fragments TDP-25 and TDP-35 appears to play a significant role in its cytoplasmic mislocalization and aggregation. Instead, overexpression of truncated TPD-25 or TDP-35 in cells is sufficient to sequester a proportion of full-length TDP-43, leading to altered RNA processing, protein homeostasis and ultimately cell toxicity (11,52). In the present study, we have characterized the propensities of TDP-25 and TDP-35 to aggregate, as compared to TDP-43, and evaluated the protective effect of the molecular chaperone HSPB8 both in motor neurones and fly models expressing TDP-43 or its truncated forms. The results first show that TDP-25 and TDP-35 fragments display different aggregation propensities, with TDP-25 being more prone to aggregation compared to TDP-35 or TDP-43. Second, they show that, in an attempt to maintain protein homeostasis and as found for other types of proteinaceous aggregates (36), TDP-25 and TDP-35 fragments are targeted to degradation by the proteasome and through autophagy. In this context, HSPB8 is a critical player in the maintenance of proteostasis, since cells overexpressing truncated TDP-25 and TDP-35 significantly upregulate endogenous HSPB8. Moreover, endogenous HSPB8 co-localizes with TDP aggregates, supporting its role in the recognition of these misfolded species. This observation is confirmed by our finding that overexpression of HSPB8 decreases the total accumulation and degree of aggregation of both TDP-25 and TDP-35 fragments, while its downregulation enhances them. Combined with our previous findings using mutated polyglutamine proteins, mutant SOD1 and ΔC-TDP-43 (15,41,46), our results generalize further the anti-aggregation function of HSPB8.

In a second series of experiments, we studied fly models expressing either mutated or truncated TDP-43 to test the protective role of HSPB8 against TDP-43 mediated toxicity *in vivo*. Since the mammalian and the *Drosophila melanogaster* (Dm) families have independently evolved, and as we had previously identified the functional ortholog of human HSPB8 in flies, called HSP67Bc (24), we used transgenic lines, either overexpressing or downregulating HSP67Bc, in order to test its potential protective effects in the flies. We found that overexpression of HSP67Bc had no significant effect on WT TDP-43 expression levels or eye phenotype in two independent TDP-43 fly models (32,33); instead, HSP67Bc significantly decreased the degree of eye degeneration caused by mutant TDP-43-NLS, which accumulates in the cytosol as a consequence of a non-functional sequence of its nuclear localization signal. Conversely, downregulation of HSP67Bc was found to enhance eye degeneration mediated by TDP-43-NLS, which correlated with its accumulation and with an increase in the total level of (poly)ubiquitinated proteins.

We found in addition that depletion of HSP67Bc increased the high MW species formed by mutant and mislocalized TDP-43-NLS, rather than the monomeric species (for which we observed a slight, but not significant, increase). Interestingly, high MW species, as well as cytoplasmic aggregates, have been found in cellular models expressing the specific mutant TDP-43-NLS that we studied here *in vivo* (30); these high MW species are ubiquitinated and resemble the species that accumulate in ALS and FTLD-U. Combined with the increase in the ubiquitinated proteins that we observed in HSP67Bc depleted flies that overexpress TDP-43-NLS, our results support a role for HSP67Bc in facilitating the removal of high MW, potentially aggregation-prone, ubiquitinated species that accumulate upon proteotoxic stress conditions such as overexpression of mutant TDP-43. This interpretation is consistent with our findings in motor neurone cells and with our previously published data demonstrating that HSP67Bc promotes autophagic flux in mammalian and *Drosophil*a S2 cells (24,28). In accord with this finding, co-expression of the *Drosophil*a sHSP CG14207, which assists the refolding of substrates in a HSP70-dependent manner, but does not promote the degradation of aggregation-prone proteins by autophagy (24,28), did not protect the fly eyes from the toxicity exerted by TDP-43-NLS, nor did it change its total levels.

The protective effect of HSP67Bc was further reinforced using a fly model expressing truncated TDP-35. Again, while HSP67Bc had no detectable effect on WT TDP-43 levels and the observed eye phenotype, its overexpression was sufficient to abolish the lethality of pupae caused by the TDP-35 fragment. We have previously characterized the toxic effects caused by overexpression of TDP-43 and TDP-25 in fly eyes and observed that both proteins caused only a mild rough eye phenotype, with no major differences in their effects on fly eye morphology (33). When comparing the toxicity exerted by TDP-43 and TDP-35, both of which were expressed from the same genomic locus, we noticed that TDP-35 expression in fly eyes resulted in a very strong toxic phenotype, abrogating fly eclosion. Thus, TDP-25 and TDP-35 fragments show different degrees of toxicity in flies, a phenomenon that could be due to their different abilities to interact with TDP-43 and to bind to RNA, since TDP-35, but not TDP-25, co-precipitates endogenous TDP-43 in cells (52) or, alternatively, to their different aggregation propensities and rates of turnover.

Finally, overexpression of HSP67Bc also protected against the eye degeneration induced by the ALS-causing mutant M337V TDP-43, further generalizing its beneficial effects to different forms of mislocalized TDP-43.

In summary, our data provide clear evidence that upregulation of HSPB8 could be an effective approach to combating those neurodegenerative diseases that are characterized by (motor) neurone loss and proteinaceous inclusions. Since aggregated TDP-43 and TDP-35 also contain RNA, and as their aggregation has an impact on RNA homeostasis, by sequestering specific TDP-43-bound RNAs (52), it is possible that HSPB8 upregulation might act not only on proteostasis but, by decreasing TDP-43 and truncated TDP-25 or TDP-35 aggregation, might also contribute to the maintenance of normal RNA homeostasis. These results, therefore, suggest that it might in the future be of great interest to examine the effects of HSPB8 on RNA homeostasis and to explore the potential protective effects of drugs that can upregulate HSPB8 expression for the treatment of neurodegenerative diseases such as ALS.

## Materials and Methods

### Chemicals

Dimethyl sulfoxide (DMSO), doxycycline and MG132 were obtained from Sigma-Aldrich (St. Louis, MO, USA), 3-Methyladenine (3-MA) was obtained from Selleckchem (Munich, Germany).

### Plasmids

pEGFP-TDP-43, pEGFP-TDP-35 and peGFP-TDP-25, coding, respectively, for the GFP-fused TDP-43 full length protein (GFP-TDP-43) and its C-terminal 35KDa (GFP-TDP-35) or 25 KDa (GFP-TDP-25) fragments, were kindly provided from Dr. L. Petrucelli (11). 2xFLAG-TDP-43, 2xFLAG-TDP-35 and 2xFLAG-TDP-25 were obtained by excising EGFP from pEGFP-TDPs plasmids (using NheI/BamHI sites) and inserting in frame a 2xFLAG sequence, flanked by NheI and BamHI restriction sites. pcDNA3 was from Life Technologies Corporation (Carlsbad, CA, USA). pDsRed-Monomer was from Clontech Lab (Mountain View, CA, USA). pCI-HSPB8 codes for human HSPB8 (53). The pcDNA5/TO-GFP-TDPs plasmids used for stable transfections were obtained by excising the GFP-TDPs fragments from pEGFP-TDPs vectors using NheI-XbaI sites and cloning into the same sites of pcDNA5/TO-NheI Vector. The pcDNA5/TO mammalian expression vector was obtained from Life Technologies Corporation and was modified to obtain the pcDNA5/TO-NheI by inserting the BamHI and NheI restriction sites into AflII and XbaI sites. Custom siRNA duplex was used to silence endogenous murine HSPB8 expression (sense: 5’-CGGAAGAGCUGAUGGUAAAUU-3’; antisense: 5’-UUU ACCAUCAGCUCUUCCGUU-3’) (Dharmacon, Thermo Scientific Life Sciences Research, Waltham, MA, USA). A custom non-targeting siRNA duplex was used as a negative control (sense: 5’-GGGUAAAGCUAGAGAGAAUUU-3’; antisense: 5’-AUUCUCU CUAGCUUUACCCUU-3’).

### Cell cultures and transfection

Immortalized mouse motor neurone cell line NSC34 (54,55) is routinely cultured in our laboratory (45,46,56–65). Cells were plated at 80,000 cells/well in 12-well multiwell plates for Western Blot (WB) and Filter Retardation Assay (FRA) analysis; for immunohistochemistry analysis, cells were plated at 35,000 cells/well in 24-well multiwell plate with coverslips. 24 h after plating, the cells were transiently transfected with lipofectamine (Life Technologies Corporation)/transferrin (Sigma-Aldrich), following the manufacture’s instructions (1 μg of plasmid DNA, 4 μl of transferrin solution and 2 μl of lipofectamine per well of 12-well multiwell plate). Inducible stably-transfected GFP-TDPs-NSC34 cell lines were obtained by transiently transfecting a TR4 NSC34 cell line (66,67), kindly provided by Dr. E. Garattini (Mario Negri Institute, Milan, Italy), with pcDNA5/TO-GFP-TDPs plasmids and selecting positive clones with 200 μg/ml hygromycin for 3 weeks. GFP-TDPs-NSC34 selected clones were cultured in low-glucose DMEM (Euroclone, Pero, Milan, Italy) supplemented with 5% of tetracycline-free serum (Euroclone), 100 μg/ml hygromycin (Euroclone) as described in Locatelli *et al*. 2012 (66). For FRA analyses, cells were plated at 70,000 cells/well in 12-well multiwell plate and induced with 1 μg/ml doxycycline for 72 h.

### Fly stocks

All fly stocks were maintained at 18 °C on a 12:12 h light:dark cycle at constant humidity on a standard Bloomington medium supplemented with yeast. For all experiments, the flies were raised at 25 °C on a 12:12 h light:dark cycle at constant humidity. Eye-specific expression of transgenes and RNAi constructs was achieved by using the GAL4-UAS system (GAL4-dependant upstream activator sequence; Brand and Perrimon, 1993). The fly stocks gmr-GAL4 (stock number 1104) and actin 5C-GAL4 (stock number 4414) were obtained from the Bloomington Drosophila Stock Centre. Transgenic flies expressing V5-HSP67Bc were constructed at Genetic Services Inc. (Sudbury, MA), and the background strain w1118 of Genetic Services was used as a control (24). RNAi lines against Dm-HSP67Bc (CG4190) were obtained from the Vienna Drosophila RNAi Center (VDRC). Two independent lines were used in this study: the VDRC ID 26416, referred to as HSP67Bc RNAi#1 and the VDRC ID 103974, referred to as HSP67Bc RNAi#2. The fly lines expressing human WT TDP-43 or the NLS mutant TDP-43 were a kind gift of Dr. JP Taylor (32). The fly lines expressing human HA-tagged TDP-43, TDP-25 or TDP-35, all incorporated at the same genomic locus (51D) on Chr. II, were generated by Bestgene Inc (USA); the fly lines expressing TDP-43 and TDP-25 have been previously described (33).

### Microscopy and immunohistochemistry

Transfected cells were fixed 48 h after transfection with 4% paraformaldeyde and methanol and processed as previously described (68). To detect endogenous and overexpressed HSPB8 protein expressions, we used a home-made rabbit polyclonal anti-HSPB8 (#25; dilution 1:200 in 5% nonfat milk) and the Alexa 594 anti-rabbit secondary antibody (A11072; Life Technologies) (dilution 1:1000 in milk). To detect FLAG-tagged TDPs, we used the anti-FLAG mouse-monoclonal antibody (F1804 Clone M2; Sigma) (dilution 1:200 in nonfat milk) and the Alexa 488 anti-rabbit secondary antibody (A11070; Life Technologies) (dilution 1:1000 un milk). Cells were stained with DAPI to visualize the nuclei. Images were acquired with LSM510 Meta system confocal microscope (Zeiss, Oberkochen, Germany) and images were processed with the Aim 4.2 software (Zeiss).

Quantification of the number of cells with GFP-TDPs or 2xFLAG-TDPs aggregates was performed on cells co-transfected with TDPs and pDSRed-monomer (used as a transfection control) by measuring the numbers of cells with TDP aggregates, normalized to the total number of transfected cells (pDSRed-monomer positive cells). At least 100 cells/field were counted, and three fields were analysed for each coverslip. The experiments were performed in triplicate and the data have been expressed in each case as mean ± sem.

### Light microscopy of fly eyes and histology

The effects of HSP67Bc overexpression or knockdown on eye degeneration in the gmr-GAL4-TDP43 WT and NLS mutant, as well as in the gmr-GAL4-TDP25 or gmr-GAL4-TDP-35 lines, were evaluated by taking light microscopic images of the eyes of 1-day old adult flies with a stereoscopic microscope model SZ40 (Olympus). For each genotype and condition more than 100 fly eyes were evaluated. For fly eye histology, heads of the appropriate genotype were collected and fixed for 2 h at room temperature in 4% paraformaldehyde in PBS. Samples were serially dehydrated in ethanol and processed as previously described (69).

### Western blot analysis, preparation of protein extracts and antibodies

Cells were harvested 48 h after transfection and centrifuged 5 min at 100 g at 4 °C. For PBS extraction, the pellets were resuspended in PBS supplemented with the protease inhibitors cocktail (Sigma-Aldrich) and homogenized using slight sonication. For NP40 extraction, the pellets were resuspended in lysis buffer (150 mM NaCl, 20 mM Trizma, NP40 0.5%, 1.5 mM MgCl_2_, glycerol 3%, pH 7.4; Sigma-Aldrich) supplemented with complete EDTA-free (Sigma-Aldrich) and 1mM dithiothreitol (DTT; Sigma-Aldrich). NP40 soluble and insoluble fractions were separated by centrifugation at 16,000 *g* for 15 min at 4 °C. The protein content of the soluble fraction was determined by the bicinchoninic acid method (BCA assay, Pierce, Rockford, IL, USA). WB analysis was performed on 12% SDS polyacrylamide gel, loading 15 μg of proteins for PBS and NP40-soluble extracts or an equal volume of the corresponding NP40-soluble fraction for NP40-insoluble extracts. Samples were electro-transferred to nitrocellulose membranes (Bio-Rad Laboratories, Hercules, CA, USA) using TransBlot Turbo Apparatus (Bio-Rad). Nitrocellulose membranes were processed as previously described (15,45,46) using the following primary antibodies: (a) rabbit polyclonal anti-GFP HRP-conjugate to detect GFP-TDPs (MB-0712; Vector Laboratories, Burlingame, CA, USA; dilution 1:10,000); (b) goat polyclonal anti-actin to detect actin (sc-1616; Santa Cruz Biotechnology; dilution 1:1000); (c) home-made rabbit polyclonal anti-HSPB8 (#25; dilution 1:1000) to detect HSPB8 (d) rabbit polyclonal anti-GAPDH to detect GAPDH (dilution sc-25778; Santa Cruz Biotechnology; 1:1000); (e) mouse-monoclonal anti-FLAG antibody to detect FLAG-tagged TDPs (F1804 Clone M2; Sigma; dilution 1:1000); (f) mouse-monoclonal anti-alpha-tubulin to detect tubulin (F2168; Sigma; dilution 1:1000). The secondary peroxidase-conjugated antibodies were: donkey anti-goat (sc-2020; Santa Cruz Biotechnology; dilution 1:10,000); goat anti-rabbit (sc-2004; Santa Cruz Biotechnology; dilution 1:10,000); goat anti-mouse (sc-2005; Santa Cruz Biotechnology; dilution 1:10,000). Signals were detected by Clarity™ Western ECL Blotting Substrate (Bio-Rad). Membranes were subsequently processed with the different antibodies after stripping for 20 min at room temperature (StripABlot, EuroClone).

Protein samples from 1- to 2-day-old fly heads were prepared by homogenizing circa 10 heads in 50 µl of 2% SDS lysis buffer. The various proteins were resolved by SDS-PAGE, transferred to nitrocellulose membrane, and then processed for WB. Mouse monoclonal α-tubulin antibody (Sigma-Aldrich) was used as a loading control and rabbit polyclonal ubiquitin antibody (DAKO) was used to detect ubiquitinated proteins in fly head extracts. Human TDP-43 in fly head extracts was detected using the TARDBP (41–7.1) antibody (sc-100871; Santa Cruz Biotechnology). The antibody against Dm-HSP67Bc has been previously described (24).

### Filter retardation assay, preparation of protein extracts

For transiently transfected NSC34 cells, 1.5 µg of the PBS or NP40-soluble protein extracts (quantified used the BCA assay as described above) were loaded onto 0.2 µm cellulose acetate membrane (Whatman, GE Healthcare) and filtered through a Bio-Dot SF Microfiltration Apparatus (Bio-Rad). An equal volume of the corresponding NP40-soluble extract was loaded in the case of NP40-insoluble extracts. Slot-blots were probed as described for WB to detect GFP-TDPs or FLAG-TDPs retained insoluble species. For stably transfected GFP-TDPs-NSC34 cell lines, cells were harvested 72 h after induction with 1 μg/ml of doxycycline, and samples were prepared as described above for transiently transfected cells. 9 µg of the NP40-detergent soluble protein extracts were loaded onto cellulose acetate membranes and filtered. An equal volume of the corresponding NP40-soluble extract was loaded in the case of NP40-insoluble extracts. Slot-blots were probed with GFP antibody as described above for WB. Densitometric optical analysis of slot-blots was performed and represented as mean ± SEM (*n =* 3).

## Statistics

Cellular biochemical data were analysed using Student’s *t*-test. Values represent mean ± SEM of 3 replicates. Fly biochemical data and eye morphological comparisons between genotypes were analysed using ANOVA, followed by the Bonferroni post-hoc or the Student’s t-test. * = *P >* 0.05; ** = *P >* 0.01; *** = *P >* 0.001. Values represent mean ± SEM.
